# Live attenuated African swine fever viruses as ideal tools to dissect the mechanisms involved in viral pathogenesis and immune protection

**DOI:** 10.1186/s13567-015-0275-z

**Published:** 2015-11-20

**Authors:** Anna Lacasta, Paula L. Monteagudo, Ángeles Jiménez-Marín, Francesc Accensi, María Ballester, Jordi Argilaguet, Iván Galindo-Cardiel, Joaquim Segalés, María L. Salas, Javier Domínguez, Ángela Moreno, Juan J. Garrido, Fernando Rodríguez

**Affiliations:** Centre de Recerca En Sanitat Animal (CReSA), Institut de Recerca i Tecnologia Agroalimentàries (IRTA), Campus UAB, Bellaterra, 08193 Barcelona, Spain; International Livestock Research Intitute (ILRI), 00100 Nairobi, Kenya; Instituto de Agricultura Sostenible, Campus Alameda del Obispo, 14080 CSIC Córdoba, Spain; Departament de Sanitat i Anatomia Animals, Universitat Autònoma de Barcelona (UAB), Bellaterra, 08193 Barcelona, Spain; INRA, UMR, 1313, Génétique Animale et Biologie Intégrative, 78352 Jouy-en-Josas, France; Universitat Pompeu Fabra, 08003 Barcelona, Spain; WorldPathol Ltd. Co., 50005 Saragossa, Spain; Centro de Biología Molecular Severo Ochoa (Consejo Superior de Investigaciones Científicas-Universidad Autónoma de Madrid), 28049 Madrid, Spain; Departamento de Biotecnologıa, INIA, 28040 Madrid, Spain; Grupo de Genómica y Mejora Animal, Departamento de Genética, Facultad de Veterinaria, Universidad de Córdoba, Campus de Rabanales, Ed. C-5, 14071 Córdoba, Spain

## Abstract

African swine fever virus (ASFV) is the causal agent of African swine fever, a hemorrhagic and often lethal porcine disease causing enormous economical losses in affected countries. Endemic for decades in most of the sub-Saharan countries and Sardinia, the risk of ASFV-endemicity in Europe has increased since its last introduction into Europe in 2007. Live attenuated viruses have been demonstrated to induce very efficient protective immune responses, albeit most of the time protection was circumscribed to homologous ASFV challenges. However, their use in the field is still far from a reality, mainly due to safety concerns. In this study we compared the course of the in vivo infection caused by two homologous ASFV strains: the virulent E75 and the cell cultured adapted strain E75CV1, obtained from adapting E75 to grow in the CV1 cell-line. Interestingly, the kinetics of both viruses not only differed on the clinical signs that they caused and in the virus loads found, but also in the immunological pathways activated throughout the infections. Furthermore, E75CV1 confirmed its protective potential against the homologous E75 virus challenge and allowed the demonstration of poor cross-protection against BA71, thus defining it as heterologous. The in vitro specificity of the CD8^+^ T-cells present at the time of lethal challenge showed a clear activation against the homologous virus (E75) but not against BA71. These findings will be of utility for a better understanding of ASFV pathogenesis and for the rational designing of safe and efficient vaccines against this virus.

## Introduction

African swine fever (ASF) is a highly pathogenic disease caused by African Swine Fever Virus (ASFV), a rather complex virus against which there is no vaccine available. Thus, eradication of the disease has relied, so far, on early diagnosis and subsequent culling of infected and exposed pigs. Only 10 years after its eradication from continental Europe, ASFV re-entered Europe arriving from Africa to the Caucasian Republic of Georgia in 2007. Since then, ASFV has spread to neighboring countries including Russia, already reaching some E.U. countries and due to the global commercialization exchanges existing today, also menacing the rest of the world [1]. The risk of endemicity in Eastern European countries has dramatically increased in recent years due to the lack of enough preparedness and resources to implement successful control programs in the affected regions with both domestic pigs and wild boars being equally affected [2]. The risk of new introductions or reintroductions of the virus in ASF-free areas is high, overall taking into account the complex epidemiological situation of ASFV in Africa [3], with more than 22 genotypes currently circulating in the area [4] and the ease with which the virus can spread [5, 6]. With these antecedents at hand, having a safe and efficient vaccine might increase the chances of controlling ASF in the future. Work done decades ago, demonstrated the failure of classically inactivated vaccine in achieving strong protection against experimental ASF infection [7–10]. Conversely, classically attenuated vaccines have been shown to be very efficient at controlling homologous experimental challenges, albeit in a rather limited way against heterologous virus challenges [11, 12]. In spite of their success, the use of live attenuated vaccines (LAV) in the field has been limited mainly due to safety problems derived from their inherent infectious nature [13]. More recently, new generation vaccines have created expectations for the future. The identification of key protective antigens within the ASFV genome [14] has allowed the possibility of attempting the design of safe DIVA vaccines for the future [15]. On the other hand, recombinant deletion of targeted virulence factors should theoretically permit the generation of efficient modified LAV against ASFV [16–18] with increased security; a field in continuous progression. Independent of their safety problems, to date LAV have provided the most relevant data regarding the mechanisms involved in ASFV-protection. Thus, today we know that both specific antibodies [19] and specific CD8^+^ T-cells [20] play crucial role in the protection afforded by LAV. Despite this, the mechanisms involved in the protection afforded by antibodies are still largely unknown and several mechanisms have been proposed [21]. Similarly, CD8^+^ T-cells have been postulated as a key T cell subset in the protection afforded by both LAV [20] and DNA vaccines [14, 15]. More advanced genomic and proteomic methodologies have opened new alternatives to better understand not only the intrinsic mechanisms involved in protection but also the early and late events implicated in viral pathogenesis. Few studies have been published so far using microarrays and almost all of them have focused on comparing the effects of ASFV strains with different virulence in vitro [22]. Albeit useful in general terms, further work is needed using well characterized homologous ASFV strains to specifically identify individual virulence factors. As above described, previous results obtained in the 80s with the attenuated E75CV1 strain [11], demonstrated the fine balance existing between death and survival after intranasal experimental infection and the capability of surviving animals to resist subsequent lethal challenge with the homologous virulent E75 virus. Here we have extended the use of the virulent E75 and its cell culture adapted counterpart E75CV1 to better compare the in vivo immunopathogenesis of both viral strains and also to understand the intrinsic mechanisms involved in protection afforded against the homologous lethal challenge. To perform these studies the optimal dose for intramuscular inoculation of E75CV1 was first standardized and next an experimental set up was designed to compare: (1) the kinetics of the E75 and E75CV1 viral infection, (2) the evolution of induced innate and specific immune responses and (3) the protective potential of E75CV1 against homologous and heterologous challenges. The sequential sampling of sera, nasal swabs, and lymphoid tissues together with a multi-technical approach allowed the further characterization of both ASFV pathogenesis and protection and has opened new avenues for further research.

## Materials and methods

### Virus strains and ethical statements

Three different ASFV strains were used in the in vivo and in vitro experiments: BA71, E75 and E75CV1. BA71 and E75 strains (both virulent) were isolated from the 1971 and 1975 Spanish ASF outbreaks, respectively. Afterwards, they were further propagated in pig leucocytes. E75CV1 isolation was achieved by 4 consecutive passages in CV1 cells (green monkey kidney fibroblasts) [11].

Experiments with 8-week-old Landrace X Large White pigs (conventional farm pigs) were performed under the approval of the Ethical and Animal Welfare Committee of the *Universitat Autònoma de Barcelona* (UAB; Permit Number: DMAH-5796) and infections with ASFV were done under biosecurity level 3 (BSL-3) conditions at *Centre de Recerca en Sanitat Animal* (CReSA, Barcelona, Spain).

### Experimental design for the in vivo experiments

Pigs were intramuscularly inoculated (i.m.i.) in the neck with 1 mL of the corresponding ASFV dose and virus strain diluted in PBS 1X pH 7.4 or with PBS 1X pH 7.4 alone as control of the assay. Animals were observed daily according to a welfare schedule to monitor their health status and to record the clinical signs after the infection with ASFV [23].

#### Experiment 1: selecting the optimal dose for E75CV1-immunization

Sixteen pigs were divided into 4 groups with 4 pigs in each one and received either PBS 1X pH 7.4 (control animals) or three different doses of the cell culture adapted strain E75CV1: 10^2^ 50% hemadsorbing units (HAU_50_), 10^4^ HAU_50_ and 10^5^ HAU_50_.

#### Experiment 2: comparative kinetics of E75 and E75CV1 infections

Fifty four pigs were divided into three groups that were maintained in isolation from each other: (a) 24 pigs were infected with the optimal dose of the attenuated E75CV1 ASFV strain, obtained from the experiment 1:10^4^ HAU_50_ and sequential blood sampling and necropsies were performed in groups of 6 pigs at days: 1, 3, 7 and 31 post-infection (pi); (b) 18 pigs were infected with the same dose (10^4^ HAU_50_) of E75, the homologous virulent virus and again groups of 6 pigs were sequentially sampled, sacrificed and subjected to necropsy at days 1, 3 and 7 pi; (c) finally, 12 animals remained uninfected and were sequentially sampled and sacrificed in groups of 3 at days 1, 3, 7 and 31 pi as controls. All animals were sampled prior to the infection (day 0 pi). During necropsies all animals and organs of interest (spleen, gastrohepatic lymph node, liver and kidneys) were weighted separately in order to establish their relative weight: percentage of the total body weight that the specific organ represents.

#### Experiment 3: homologous versus heterologous ASFV-challenge

Sixteen pigs were divided in two groups housed separately, from this point on termed BOX-1 and BOX-2 respectively. Four pigs from each room were i.m.i. inoculated with 10^4^ HUA_50_ of the E75CV1 (optimal dose of immunization selected in experiment 1), and the other four from each box remained uninfected receiving the same volume of PBS 1X pH 7.4. Twenty-eight days after E75CV1-inoculation all pigs from BOX-1 were challenged with 10^4^ HAU_50_ of the homologous E75 strain, and pigs from BOX-2 were challenged with 10^3^ HAU_50_ of BA71; corresponding to 20 lethal dose fifty (LD_50_) for each one of the ASFV strains ([24] and unpublished data).

### Virus titration

Blood samples were collected before and at different times after virus challenge for viremia quantification using the haemadsorption technique, as previously described [14, 25]. Titres were calculated by the Reed and Muench method [26] and expressed as HAU_50_/mL.

A real time quantitative PCR (qPCR) method recently described in our laboratory [15], was used to quantify the viral DNA from nasal swabs-PBS suspensions and tissues (gastrohepatic lymph node). Briefly, the viral DNA was obtained from 200 μL of swab-PBS suspensions using the NucleoSpin blood kit or from 25 mg of tissue using NucleoSpin tissue kit (both from Macherey–Nagel, Düren, Germany) following the manufacturer’s recommendations and used as template to amplify an 85 bp-long fragment from the ASFV serine protein kinase gene (*R298L*). PCR amplifications were performed in triplicate using the corresponding standards for absolute quantification. The results were expressed as log_10_ genome equivalent copies (GEC) per mL of nasal swab.

Additionally ASFV-infected macrophages were double-stained using the antibodies anti-CD172a (hybridoma clone BA1C11), specific for monocytes and macrophages and anti-p30 (hybridoma clone 1D9), specific for the p30 virus structural protein, and after secondary staining, the cells were FACS-analyzed [15].

### Histopathology and microscopic tissue evaluation

Tissue samples (spleen, gastrohepatic lymph node, liver and kidneys) were previously fixed in 10% formalin (Sigma-Aldrich, Madrid, Spain) and embedded in paraffin-wax for histological processing. Four μm-sections were stained with haematoxylin and eosin for microscopical examination. Infiltration of macrophages, apoptosis and ASFV-antigen detection were evaluated by specific immunohistochemical methods as briefly described below. Tissue sections were incubated for 30 min in a 3% hydrogen peroxide solution (diluted in absolute methanol) to inhibit the endogenous peroxidase. Afterwards, the sections were treated with EDTA (0.37 mg/mL, diluted in PBS 0.5% Tween20, pH 8) at 98 °C for 15 min and then blocked with 2% bovine serum albumin (BSA, Sigma-Aldrich, Madrid, Spain) for 1 h at room temperature for further specific antibody labeling. Both, primary and secondary antibodies were incubated for 18 h at 4 °C and subsequently treated with the avidin–biotin complex solution (ABC, Thermo Scientific, Rockford, IL, USA); labeling was revealed with 3,3′-diaminobenzidine (DAB, Sigma-Aldrich, Madrid, Spain).

Infiltrated macrophages were detected using the anti-MAC387 rabbit-antibody (1:40, Dako, Barcelona, Spain) and apoptotic cells were detected using an anti-caspase-3 rabbit-antibody (1:100, Cell Signaling Technology, Barcelona, Spain). In both cases the secondary antibody used was an anti-rabbit IgG peroxidase conjugated (1:100, Dako, Barcelona, Spain). ASFV-infected cells were detected using an anti-p30 antibody (undiluted clone 1D9) generously provided by INIA (Madrid) and using as a secondary antibody an anti-mouse IgG peroxidase-conjugated antibody (1:200, Dako, Barcelona, Spain).

### Measuring specific antibody responses

ASFV specific antibodies in pig sera were detected by the OIE-approved ELISA assay based on the use of soluble extracts from ASFV infected cells [27]. The presence of positive sera was detected using 100 μL of peroxidase-conjugated anti-pig IgG at 1/20 000 dilution (Sigma-Aldrich, Madrid, Spain) as secondary antibody and 50 μL of soluble 3,3′,5,5′-tetramethylbenzidine (TMB) as specific peroxidase substrate (Sigma-Aldrich, Madrid, Spain). Reactions were stopped with 50 μL of 1 N H_2_SO_4_ (Sigma-Aldrich, Madrid, Spain) per well. The plates were read at 450 nm wavelength (λ_450_) and the results were represented as the average absorbance (optical density (OD) values) between duplicates. Additionally, sera were used to test their ability to inhibit the in vitro infection of E75 in porcine alveolar macrophages following slightly modified previously described protocols [28]. Briefly, serial dilutions of sera (1:10 and 1:20) were first complement-inactivated at 56 °C for 30 min. After inactivation the sera dilutions were incubated with 4 × 10^5^ HAU of E75 for 1 h at 37 °C (gently mixed every 15 min), prior to the infection of porcine alveolar macrophage monolayers (4 × 10^5^ cells/well in a 96-well plate) for 72 h. The ASFV titres were finally expressed as HAU_50_/mL by using the haemadsorption technique [31].

### Measuring specific T-cell responses

Peripheral blood mononuclear cells (PBMCs) were isolated from whole blood by density-gradient centrifugation with Histopaque 1077 (Sigma-Aldrich, Madrid, Spain). For PBMC cultures, RPMI 1640 medium supplemented with 10% fetal calf serum (Invitrogen, Barcelona, Spain), 50 000 IU penicillin/l (Invitrogen, Barcelona, Spain) and 50 mg streptomycin/l (Invitrogen, Barcelona, Spain) was used. Trypan blue was used to assess cell-viability. The frequency of ASFV-specific IFNγ-secreting cells (IFNγ-SC) in PBMC was analyzed by an ELISPOT assay using commercial mAbs (Swine IFNγ, Cytoset; Biosource Europe) following a previously reported method [15]. PBMCs were specifically stimulated in vitro with 10^6^ HAU_50_/mL of the E75 ASFV strain for 20 h and 10 µg/mL Phytohemaglutinin (PHA, Sigma-Aldrich, Madrid, Spain) was used as positive control for the assay. To calculate the frequencies ASFV-specific IFNγ-SC, counts of spots in unstimulated wells were subtracted from counts in virus-stimulated wells. The frequency of cytokine-producing cells was expressed as responding cells in 10^6^ PBMCs.

Alternatively, PBMCs were labeled with carboxyfluorescein diacetate succinimidyl ester (CFSE, Vybrant CFDA SE Cell Tracer kit, Invitrogen, Barcelona, Spain), in vitro stimulated with 10^6^ HAU_50_/mL of either the E75 or the BA71 ASFV strains for 72 h, and finally cell viability was assessed by staining with LIVE/DEAD^®^ Fixable Violet Dead Cell Strain Kit (Invitrogen, Barcelona, Spain) and surface-labeled with anti-CD8-AlexaFluor647 (clone 76-2-11) and anti-CD4-PerCPCy5.5 (clone 74-12-4) antibodies (BD Pharmingen, NJ, USA), allowing the phenotyping of the virus-specific proliferating cells [29]. Combining these two markers, three different T-cell populations could be defined [30]: CD4^+^ T-cells (single positive CD4), cytotoxic T-cells or CTLs (single positive CD8^high^) and memory T-cells (double positive CD4^+^CD8^low^).

### Analysis of relative gene expression by qPCR

After necropsy, gastrohepatic lymph node samples were aseptically collected, overnight treated with RNA*later*^®^ (Ambion, Austin, TX, USA) at 4 °C to increase the RNA stability of the tissues and next frozen at −80 °C for further RNA isolation. RNA was isolated using the RNeasy Mini kit (Qiagen, Valencia, CA, USA), treated with RNase-free DNase Set (Qiagen, Valencia, CA, USA) and impurities removed with RNeasy Minielute kit (Qiagen, Valencia, CA, USA), following the manufacturer’s instructions. The integrity, quality and quantity of RNA obtained was checked in 1% (w/v) agarose gels and using a NanoDrop™ 1000 Spectrophotometer (Thermo Scientific, Rockford, IL, USA). qPCR technology was used to determine the relative expression of a panel of 19 swine gene transcripts representative of Th1, Th2 and Th17 responses (Table 1), previously described [31, 32]. One μg of RNA isolated from infected and control animals at different times post-infection was reverse transcribed to cDNA using the iScript cDNA Synthesis kit (BioRad, Hercules, CA, USA). Three animals from each time point and group were selected taking into account the quality and quantity of the RNA obtained. A PCR specific for the 18S was performed with all cDNAS in order to assess their quality. Final concentration of primers in the PCR reactions was 0.5 μM and primers (Table 1) were designed using Beacon Designer™ (Biosoft International, Palo Alto, CA, USA) and internal normalization of the gene expression analysis was carried out by using beta-actin and *PPIA* as reference genes [33]. The relative gene expression was assessed by the 2^−ΔΔCt^ method [34] and applied as previously described [31]. With this method, a fold-change value of 1 or −1 represents no difference in gene expression. The differences in mRNA expression among groups were assessed by the Student’s paired T-test using SigmaPlot v10.0 (Systat Software Inc., CA, USA). Values of *p* < 0.05 were considered as being significant.

**Table 1:** List of genes and sequences of the primers used for qPCR analysis.

Gene name	Forward primer (5′ → 3′)	Reverse primer (5′ → 3′)	Accession number
*β*-*actin*	CAGGTCATCACCATCGGCAACG	GACAGCACCGTGTTGGCGTAGAGGT	U07786
*PPIA*	CCTGAACATACGGGTCCTG	AACTGGGAACCGTTTGTGTTG	AY266299
*18S*	GACTCAACACGGGAAACCTCAC	GCTTATGACCCGCACTTACTGG	AY265350
*TNFα*	CCTCTTCTCCTTCCTCCTG	CCTCGGCTTTGACATTGG	X57321
*IL*-*1β*	GGCCGCCAAGATATAACTGA	GGACCTCTGGGTATGGCTTTC	NM_214055
*IL*-*4*	TTGCTGCCCCAGAGAAC	TGTCAAGTCCGCTCAGG	AY294020
*IL*-*5*	TGGTGGCAGAGACCTTGACA	CCATCGCCTATCAGCAGAGTT	NM_214205.1
*IL*-*6*	TGGCTACTGCCTTCCCTACC	CAGAGATTTTGCCGAGGATG	NM_214399
*IL*-*8*	TTCGATGCCAGTGCATAAATA	CTGTACAACCTTCTGCACCCA	AB057440
*IL*-*10*	CAGATGGGCGACTTGTTG	ACAGGGCAGAAATTGATGAC	L20001
*IL*-*12p40*	GGAGTATAAGAAGTACAGAGTGG	GATGTCCCTGATGAAGAAGC	U08317
*IL*-*13*	AAGTGGCCCAGTTCGTAAAAGA	ACCCGTGGCGAAAAATCA	NM_213803.1
*IL*-*17A*	CTCTCGTGAAGGCGGGAAT	TCCTCAGTTTTTGGGCATCCT	NM_001005729
*IL*-*18*	AGGGACATCAAGCCGTGTTT	CGGTCTGAGGTGCATTATCTGA	EU118362.1
*IL*-*21*	CTGCCTGATGGTCATCTTCTCA	AGGCGATCTTGTCCTTGGAA	NM_214415
*IL*-*23*	GCTTGCAAAGGATCCACCAA	GGCTCCCCTGTGAAAATGTC	NM_001130236.1
*IFN*-*γ*	CAAAGCCATCAGTGAACTCATCA	TCTCTGGCCTTGGAACATAGTCT	X53085
*TGF*-*βR1*	TCAGGCTTACCATTGCTTGTTCAG	CTCGCCAAACCTCTCCAAATCG	NM_001038639
*DEFB1*	ACCGCCTCCTCCTTGTATTC	GGTGCCGATCTGTTTCATCT	NM_213838
*DEFB2*	CTGTCTGCCTCCTCTCTTCC	CAGGTCCCTTCAATCCTGTT	NM_214442
*NF*-*κB*	CTCGCACAAGGAGACATGAA	ACTCAGCCGGAAGGCATTAT	DQ834921
*CD163*	GCCTGTCTCATCGCATTCCT	GGATTTAGCATATCCGTTTCATCTG	NM_213976.1

### ELISA detection of serum soluble factors

Serum samples were harvested in polypropylene tubes and stored at −80 °C until use. ELISA was used for serum interferon-α (IFN-α, R&D, Minneapolis, MN, USA) and tumour necrosis factor-α (TNF-α, R&D, Minneapolis, MN, USA), according to the manufacturer’s instructions. Respective standard curves were used to determine the amounts (U/mL in IFN-α assay and pg/mL in TNF-α assay) of each cytokine in the porcine serum samples. IFN-α and TNF-α amounts were measured both in control and infected pigs at each sampling time. Soluble CD163 (sCD163) was quantified in sera by a previously described enzyme-linked immunosorbent assay (ELISA), using lysates from CD163-transfected CHO cells as a standard, and results were expressed as the equivalent number of CD163-transfected cells [35].

## Results

### E75CV1, an ideal experimental infection model but not a safe vaccine prototype

**Figure 1:**
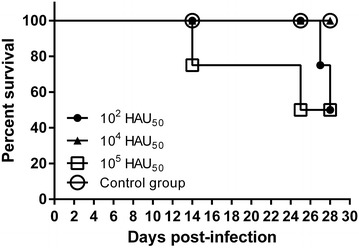
**Percentage of surviving animals (experiment 1).** Groups of four pigs were inoculated intramuscularly with different doses of the cell culture adapted strain E75CV1: 10^2^ HAU_50_, 10^4^ HAU_50_ and 10^5^ HAU_50_; and the control group.

**Figure 2:**
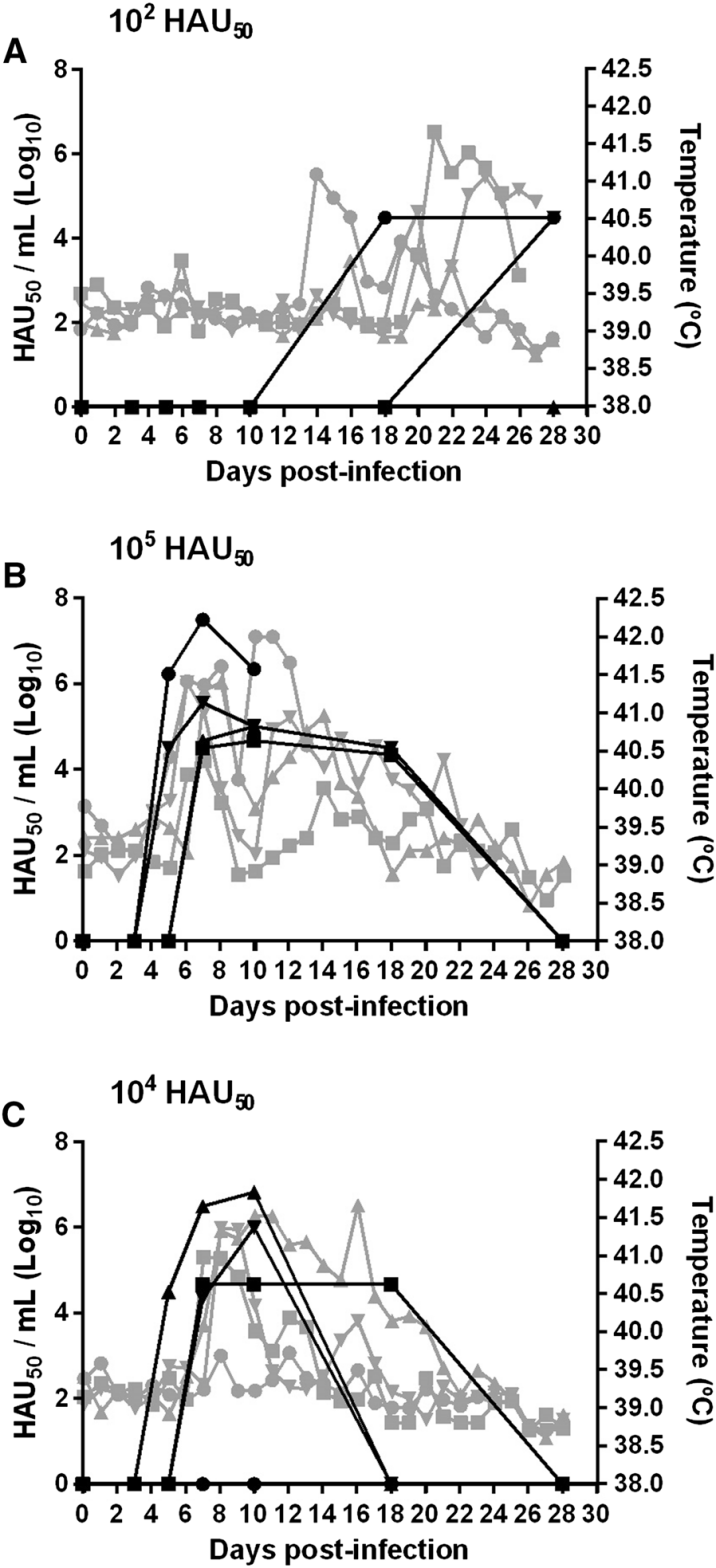
**ASFV-viremia and fever kinetics (experiment 1).** Groups of four pigs were intramuscularly inoculated with different doses of the cell culture adapted strain E75CV1: 10^2^ HAU_50_ (**A**), 10^5^ HAU_50_ (**B**) and 10^4^ HAU_50_ (**C**) and both viremia and rectal temperature was recorded throughout the infection. ASFV-Virus detection in individual sera was quantified by hemadsorption (black lines) and daily rectal temperature was also measured (grey lines).

**Figure 3:**
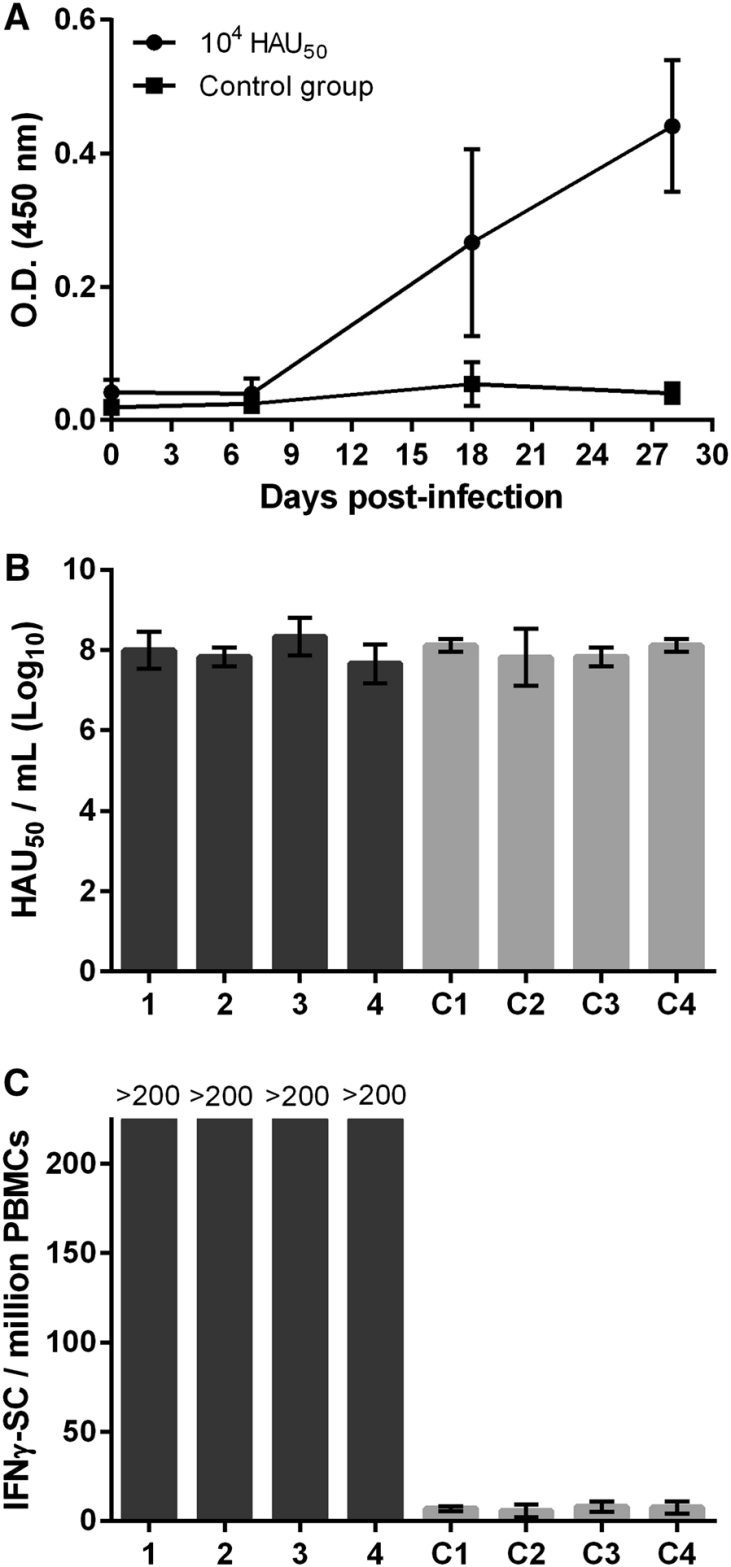
**Specific immune responses induced by E75CV1 (experiment 1).** Two groups of four pigs were intramuscularly inoculated with 10^4^ HAU_50_ E75CV1 or were left as non-immunized controls. The kinetics of the individual specific antibody induction was monitored by ELISA. Average and standard deviation values are shown (**A**). At day 28 pi sera from the 10^4^ HAU_50_ immunized animals were additionally subjected to an infection inhibition assay in vitro (**B**) and ASFV-specific T-cells were measured at day 28 pi by an IFNγ-ELISPOT assay. Average values and standard deviation shown correspond to three individual experiments for the E75CV1-vaccinated (1–4) and control animals (C1–C4) (**C**).

Due to the complexity of standardizing a dose for E75CV1 intranasal immunization, the intramuscular route for E75CV1 in vivo inoculation was tested (Experiment 1). Therefore, 3 groups of four pigs each were i.m.i. with either 10^2^ HAU_50_, 10^4^ HAU_50_ or 10^5^ HAU_50_ of the E75CV1 strain and deaths were recorded during the course of the infection. Interestingly enough, all pigs i.m.i. with 10^4^ HAU_50_ of E75CV1 survived the infection, while 2 out of the 4 pigs (50%) that received ten times more virus (10^5^ HAU_50_ of E75CV1), died at days 14 and 25 pi, respectively (Figure 1) with clear clinical signs of acute ASF. Intriguingly, infection with 10^2^ HAU_50_ E75CV1 (100 times lower than the intermediate dose), also resulted in an unexpected course of the disease, which resolved with the death of 2 out of the 4 pigs by days 27 and 28 pi (Figure 1). The 2 surviving pigs showed no detectable viremia and very short peaks of mild fever while fatal cases coincided with the first appearance of ASF clinical signs by day 17 pi, correlating with the detection of viremia and fever (Figure 2A). Conversely, pigs succumbing after the challenge with 10^5^ HAU_50_ of E75CV1 showed viremia and fever that peaked as early as day 5 pi, 3 days before the surviving animals that also showed lower and shorter viremia and fever peaks (Figure 2B). Finally, i.m.i. with 10^4^ HAU of E75CV1 resulted optimal not only because there were no deaths (Figure 1) but also because pigs infected with this intermediate virus dose showed either no viremia and no clinical signs of acute ASF (in one of the pigs) or just limited viremia and fever episodes that reached undetectable levels by day 28 pi (Figure 2C), indicating a total recovery from ASFV infection. The detection of ASFV-specific antibodies from day 18 pi by ELISA (Figure 3A) and T-cells by IFNγ-ELISPOT at day 28 pi (Figure 3C), clearly demonstrates the efficient infection of every single pig i.m.i. with 10^4^ HAU of E75CV1, including pig 1 that showed no viremia (Figure 2C), or ASF-compatible signs throughout the duration of the experiment (data not shown). In spite of the high titres of specific antibodies induced, sera from these animals were not capable to inhibit the ASFV-infection in alveolar macrophages (Figure 3B).

### E75CV1/E75, an ideal model for the characterization of ASFV immunopathogenesis

**Figure 4:**
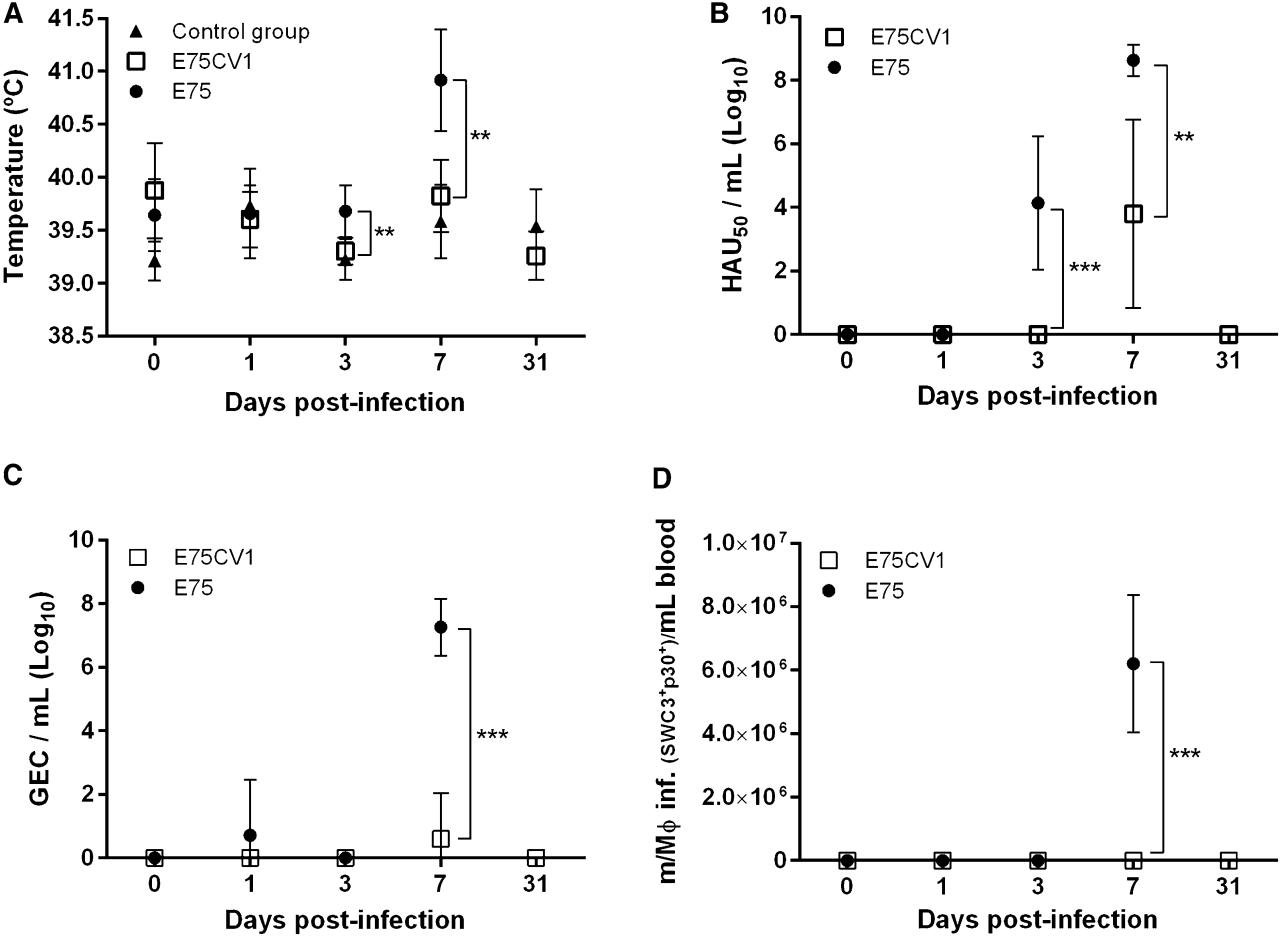
**Comparative in vivo infection with virulent (E75) and attenuated (E75CV1) homologous ASFV-strains (experiment 2).** ASFV infection was monitored over time by means of rectal temperature (**A**), viremia as measured by haemadsorption (**B**), nasal viral excretion by means of qPCR (**C**) and number of infected monocytes found in blood by cytometry (**D**) The graphs show average values and standard deviations per six-animal group (**p < 0.01 and ***p < 0.001).

**Figure 5:**
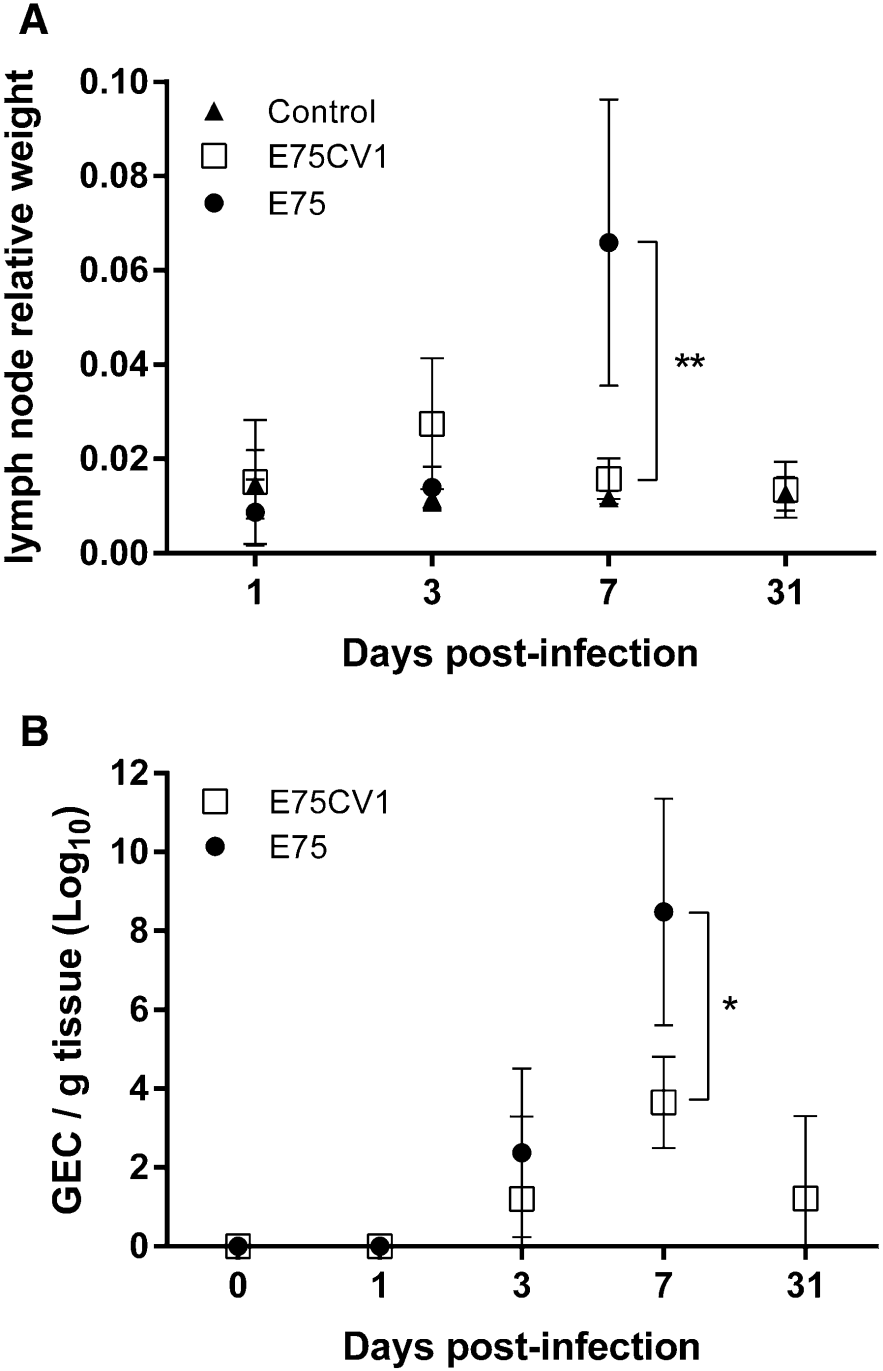
**Gastrohepatic lymph nodes and ASFV-infection (experiment 2).** Relative gastrohepatic lymph node weight versus total body weight (**A**) and ASFV-titers found in gastrohepatic lymph node by means of qPCR (**B**). The figures show the average and the standard deviation of each six animal group (*p < 0.05 and **p < 0.01).

**Figure 6:**
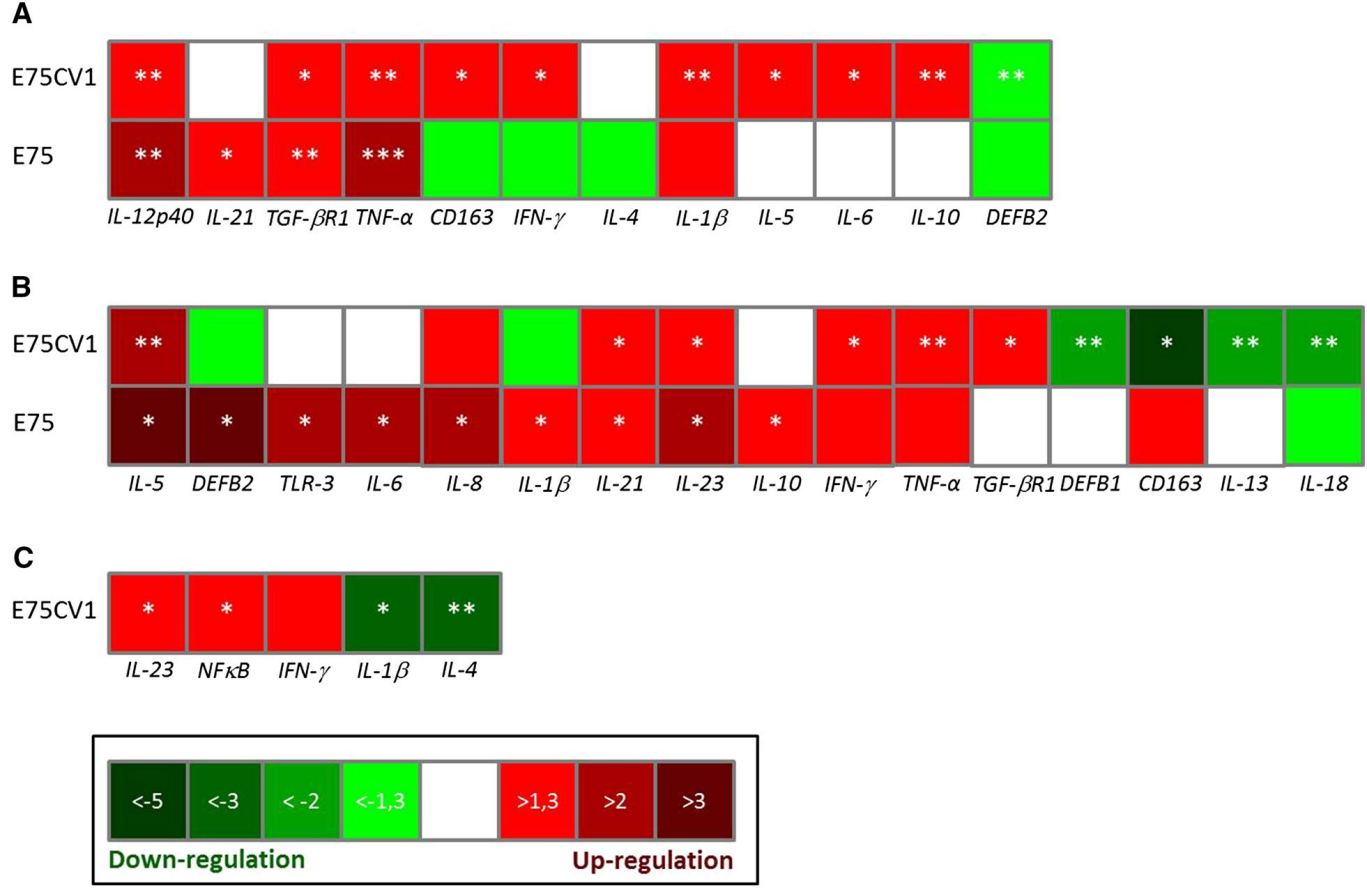
**Relative gene expression analysis in gastrohepatic lymph node of ASFV-infected pigs (experiment 2).** Statistical analysis (using T-Student test: *p < 0.05; **p < 0.01 and ***p < 0.001) comparing the RNA expression levels found for key regulatory molecules in gastrohepatic lymph nodes of animals infected with E75CV1 (attenuated) or E75 (virulent) homologous ASFV strains, relative to non-infected animals (control group). Values corresponding to three pigs per group and times are represented: 1 day (**A**), 7 days (**B**) and 31 days (**C**) post-infection. The colour grade reflects the fold-change differences found (the higher the transcription up or down regulation, the darker the colour): the green gradient represents transcription down-regulation (from −1.3 to −fivefold changes) and the red gradient represents transcription up-regulation (from 1.3–threefold changes). No colour (white) indicates no changes in the corresponding transcript level. No survivors from the E75 ASFV inoculated pigs were available at day 31 pi.

In spite of its advantages, determining viral kinetics and the immunological responses induced throughout the infection by sequentially sampling (blood and nasal swabs) individual pigs, also have important limitations especially when considering full characterization of the in vivo pathogenesis of ASFV, a virus which mainly targets the lymphoid tissues. Therefore, a complex in vivo experiment was designed (Experiment 2) aiming to compare ASFV pathogenesis of cell culture adapted E75CV1 and its virulent homologous counterpart, E75. Taking advantage of the optimization of the intramuscular route for E75CV1 in vivo inoculation (Experiment 1), a group of 24 pigs was i.m.i. with 10^4^ HAU_50_ of E75CV1, a second group of 18 pigs was infected using the same dose and route of the virulent E75 and finally a third group of 12 pigs remained uninfected (control group). Pigs from the three groups were sacrificed at days 1, 3 and 7 pi (6 or 3 per group and day) and 6 pigs from the attenuated E75CV1 and 3 from the control group were also sacrificed at day 31 pi. As expected for the virus and dose used [23], pigs infected with E75 developed severe ASF clinical signs, evident from day 3–5 pi, including: fever (Figure 4A), dyspnoea and depression. In contrast, the animals infected with the attenuated isolate (E75CV1) only developed, if at all, slight transient fever (Figure 4A). The viremia (Figure 4B), the viral excretion (Figure 4C) and the infected macrophages counts in blood (Figure 4D) evolved in correlation with these observations. The animals infected with the attenuated isolate suffered a delay in the virus detection in sera (Figure 4B), in nasal excretion (Figure 4C) and in the infected macrophages counts in blood (Figure 4D), detectable only at day 7 pi and the virus titres were 4–5 logs lower on average than those found in pigs infected with the E75 virulent strain, reaching up to 10^9^ HAU_50_/mL in sera. The lesions found after necropsy, mostly in E75-infected pigs and taking into account the macro and micro organ structure, relative organ weight (Figure 5A), virus titres found (Figure 5B), the presence of cellular infiltrations, apoptosis and viral presence (Table 2), confirmed gastrohepatic lymph node as the organ of choice to further compare the immunopathogenesis of both virus strains. Total RNA isolation from gastrohepatic lymph nodes obtained from each animal and day of study allowed the establishment of a comparative transcription profile of key immune mediators that were differentially modulated throughout the infections. Interestingly, differences became evident as early as at day 1 pi, before the virus was detectable in the tissue (Figure 5B). Thus, compared with the 10 genes significantly up and down-regulated by day 1 pi with E75CV1, E75 only up-regulated 4, namely: *IL*-*12p40*, *TGF*-*βR1*, *TNF*-*α* and *IL*-*21*, with key activation markers such as s*CD163*, *IFN*-*γ* and *IL*-*4* showing tendency towards being down-regulated (Figure 6A). This tendency totally reverted through the infection course and by day 7 pi the activation of a massive number of immune mediators was observed as demonstrated in here with the significant up-regulation of: *IL*-*5*, *DEFB2*, *TLR*-*3*, *IL*-*6*, *IL*-*8*, *IL*-*1β*, *IL*-*21*, *IL*-*23* and *IL*-*10*; coinciding with the cytokine storm described during the terminal phase of acute ASF [36] (Figure 6B). The transcription profile observed in the gastrohepatic lymph nodes of E75CV1-infected pigs showed an inverted cytokine response when compared to that of E75. Converse to that described for E75, E75CV1 efficiently activated an innate immune response detectable at day 1 pi with the significant up-regulation of *CD163*, *IL*-*1β*, *IFN*-*γ*, *IL*-*5*, *IL*-*6*, *IL*-*12p40*, *TNF*-*α*, *IL*-*10* and *TGF*-*βR1*; most probably contributing to control of the first rounds of E75CV1-replication (Figure 6A). Confirming the inverse tendency described for E75, a more equilibrated immunological balance was observed with the progression of the E75CV1-infection, with 6 genes showing significant transcription up-regulation at day 7 pi: *IFN*-*γ*, *IL*-*5*, *TNF*-*α*, *TGF*-*βR1*, *IL*-*21*, *IL*-*23* and 4 extra genes showing a significant down-regulation: *DEFB1*, *CD163*, *IL*-*13* and *IL*-*18* (Figure 6B). Finally, pigs surviving the infection with E75CV1 showed a very consistent signature characterized by the up-regulation of *IL*-*23*, *IFN*-*γ* and *NFκB* and the down-regulation of *IL*-*1β* and *IL*-*4* (Figure 6C).

**Table 2:** **Histopathology evaluation (experiment 2)**. Quantification of macrophage infiltrations (A), presence of apoptotic cells (B) and ASFV antigen detection (C) in gastrohepatic lymph node.

	Animal	Day 1 pi	Day 3 pi	Day 7 pi	Day 31 pi
(A)					
Macrophage infiltration (MAC387^+^)					
E75	1	+	+	+++	
	2	+	+	+++	
	3	+	+	++	
E75CV1	1	+	+	+	+
	2	++	++	++	+
	3	++	+	++	+
Control	1	+	+	+	+
	2	+	+	+	+
	3	+	+	+	+
(B)					
Apoptotic cell quantification (Caspase-3^+^)					
E75	1	+	+	+++	
	2	+	+	++	
	3	+	+	++	
E75CV1	1	+	+	+	+
	2	+	++	+	+
	3	+	+	+	+
Control	1	+	+	+	+
	2	+	+	+	+
	3	+	+	+	+
(C)					
ASFV Antigen detection (p30^+^)					
E75	1	+	++	+++	
	2	–	+	++	
	3	–	++	+++	
E75CV1	1	+	–	+	+
	2	–	–	+	+
	3	+	+	–	++

Results were quantified as follows: (–) no detection, (+) low, (++) moderate and (+++) high amounts

### E75CV1, an ideal model to understand the mechanisms involved in protection against homologous lethal challenge

Once the optimal dose of E75CV1 i.m.i. was confirmed (Experiment 1), a final experiment was performed to evaluate its protective potential (Experiment 3). Confirming the results obtained in experiments 1 and 2, all pigs (8 out of 8, 4 animals per box) i.m.i. with 10^4^ HAU of E75CV1 survived the infection without significant ASF clinical signs and showing none or low fever and viremia peaks that resolved by day 28 pi, coinciding with the presence of a high level of ASFV-specific antibodies and a large number of ASFV-specific T-cells as measured by ELISA and IFNγ-ELISPOT, respectively (data not shown). At this time point, all pigs in BOX-1 were challenged with a lethal dose of E75 and all animals in BOX-2 with a lethal dose of the BA71 virus strain.

**Figure 7:**
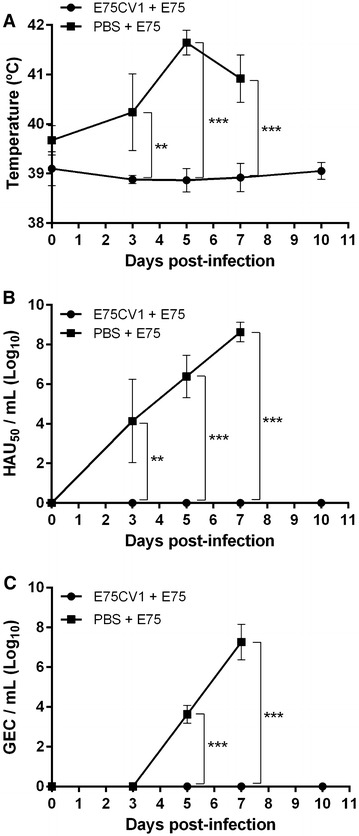
**Inoculation of E75CV1 confers protection against homologous E75 lethal challenge (Box 1 from experiment 3).** Groups of four pigs were either inoculated with 10^4^ HAU_50_ E75CV1 or non-immunized (control) and 31 days later were challenged with a lethal dose of 10^4^ HAU_50_ of E75. The ASFV infection was montitored by: rectal temperature (**A**), virus detection in sera by hemadsorption (**B**) and nasal excretion by means of qPCR (**C**). The graphs show the average values of each group and the standard deviations (**p < 0.01 and ***p < 0.001).

**Figure 8:**
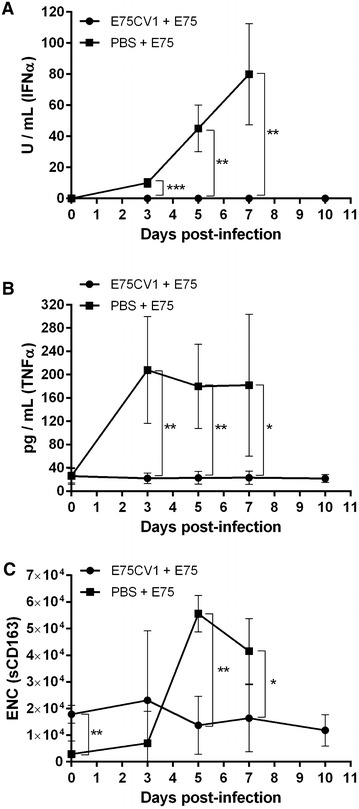
**Inoculation of E75CV1 protects against the cytokine storm provoked by homologous E75 lethal challenge (Box 1 from experiment 3).** Groups of four pigs were either inoculated with 10^4^ HAU_50_ E75CV1 or non-immunized (control) and 31 days later were challenged with a lethal dose of 10^4^ HAU_50_ E75. The delivery of cytokines and soluble cellular factors to the sera of the infected pigs was measured throughout the ASFV-challenge by specific ELISAs for: IFN-α (**A**), TNF-α (**B**) and sCD163 (**C**). Graphs show average values and standard deviations per group (*p < 0.05; **p < 0.01 and ***p < 0.001).

E75CV1 infected animals rendered 100% of protection against the homologous E75 lethal challenge and importantly, the protection afforded was complete since no ASF-clinical signs including fever (Figure 7A), viremia (Figure 7B) and nasal viral excretion (Figure 7C) were observed at any time in any of the inoculated animals. Additionally, no ASF-compatible lesions were detected in any of the animals at necropsy, while control animals behaved as expected: severe signs of ASF, severe ASF-compatible lesions found at necropsy time and high virus titres (in blood and nasal swabs) detectable from day 3 pi (Figures 7A, B and C). The control of the infection by pigs vaccinated with 10^4^ HAU_50_ of E75CV1 correlated with the control of the cytokine storm typically triggered by virulent ASFV strains such as E75. Thus, the levels of IFN-α (Figure 8A) and TNF-α (Figure 8B) in pig sera remained at very low or undetectable levels in protected animals, while control animals showed an exponential increase in the sera levels of both cytokines after lethal challenge with the homologous E75 virulent ASFV strain, as was previously described, at least for TNF-α [37]. Besides these two cytokines, several other soluble factors were found at high levels in sera from severely ASF-affected pigs by day 7 pi with E75, including IL-12 and IL-1β (data not shown) and for the first time, also for sCD163 (Figure 8C), an activation macrophage marker that has been associated with several chronic inflammatory diseases, sepsis, and more recently also with certain haemorrhagic fever diseases caused by viruses [38].

**Figure 9:**
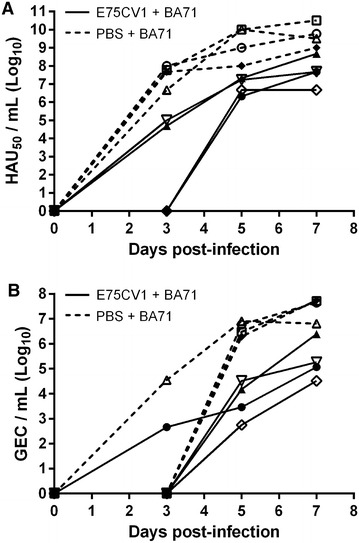
**Inoculation of E75CV1 does not confer protection against heterologous BA71 lethal challenge (Box 2 from experiment 3).** Groups of four pigs were either inoculated with 10^4^ HAU_50_ E75CV1 (solid lines) or non-immunized (control, discontinuous lines) and 31 days later were challenged with a lethal dose of 10^3^ HAU_50_ BA71. The ASFV infection was monitored by: ASFV detection in sera by hemadsorption (**A**) and nasal excretion by means of qPCR (**B**). The graphs show the values corresponding to individual pigs.

Conversely, pre-immunization with E75CV1 rendered poor protection against BA71 lethal challenge. Two of the animals (50%) reached viremia levels (Figure 9A) and nasal viral excretion titres (Figure 9B) indistinguishable from those found in control pigs, while the other 2 pigs showed a delay in the appearance of viremia (from day 3 to day 5 pi), reaching lower levels comparing with control group. Moreover, all the animals suffered from severe clinical signs of ASF, being sacrificed at day 7 pi for ethical reasons. At the time of necropsy it was not possible to differentiate the controls and the vaccinated animals (data not shown).

**Figure 10:**
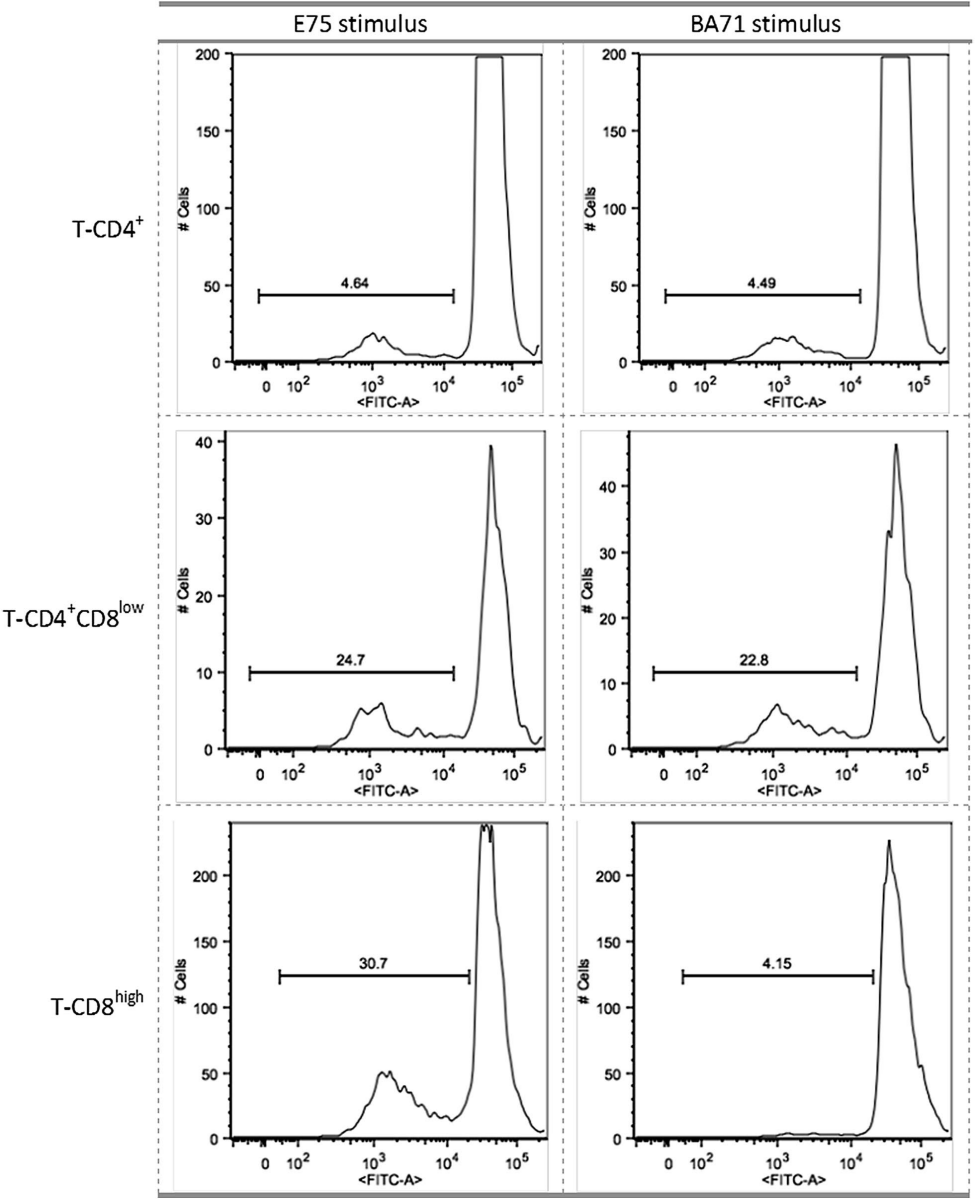
**The presence of specific CD8** ^**+**^ **T cells capable of in vitro proliferation in response to a specific ASFV strain correlates with in vivo protection**. Detection of ASFV-specific CD4^+^, double positive CD4^+^/CD8^low^ or CD8^high^ proliferating T-cells of E75CV1-inoculated pigs at day 28 pi, just before ASFV-virulent challenge. The specific proliferation was performed by labeling the PBMCs with CFSE followed by in vitro stimulation with either E75 or BA71 ASFV strains as stimuli. PBMCs were finally stained with anti-CD4 or anti-CD8 antibodies and analysed by flow cytometry. The percentage of specific CD4^+^, double positive CD4^+^/CD8^low^ and CD8^high^ proliferating T-cells is also shown.

Intriguingly, sera and PBMCs isolated from pigs surviving E75CV1 (day 28 pi), equally recognized E75 and BA71 in vitro, at least using the home-made ELISA and IFNγ-ELISPOT assays and again, sera from these animals were not capable of inhibiting the ASFV-infection in alveolar macrophages (data not shown). Aiming to further explore the mechanisms that could explain E75-homologous protection and the lack of cross-protection against BA71, the PBMCs from animals infected with E75CV1 at day 28 pi were subjected to an in vitro CFSE-proliferation assay using either E75 or BA71 as specific stimuli for three days. Interestingly, immunization with the attenuated E75CV1 induced double positive CD4^+^/CD8^low^ T-cells capable of specifically recognizing both viruses, E75 and BA71 (Figure 10). In clear contrast, their single positive CD8^high^ T-cells specifically proliferated almost exclusively in response to the homologous E75 virus, with the heterologous BA71 provoking a poor CD8^+^ T-cell stimulation (Figure 10). A poor stimulation was also observed in response to both viruses for the single positive CD4^+^ T-cells (Figure 10).

## Discussion

The fine balance observed between surviving and death after ASFV infection seems to rest on multiple factors, including the pathogenicity of a particular ASFV strain, the initial viral dose received by the pig and the health status of the particular animal. Thus, receiving ten times more ASFV than the optimal dose of the cell culture adapted E75CV1 provoked the death of 50% of the pigs, corresponding with their incapability to control the initial rounds of replication as demonstrated with the detection of early viremia from day 5 pi, 3 days before surviving pigs. The fact that infection with 10^4^ HAU_50_ of the virulent E75 strain also coincided with early viremia detectable from days 3–5 pi, correlating with an impaired recognition by the immune system very early after the infection (see below), allows us to hypothesize that a similar scenario could also explain the fate between surviving and death after i.m.i. with high doses of E75CV1 (2 out of 4 pigs dying after i.m.i. with 10^5^HAU_50_ of the virus). In agreement with this hypothesis, the viremia and the clinical signs observed and the postmortem lesions found in these two pigs were indistinguishable from those occurring after E75-infection. The unexpected results obtained after inoculating pigs with the lower dose of E75CV1 (100 HAU_50_) might be explained by direct in contact-transmission, most probably with the two pigs succumbing to the high infectious-dose of E75CV1. Even if this was the scenario, it accurately reflects what might occur under field conditions, with a proportion of naïve pigs becoming severely infected by exposure to large amounts of a live attenuated vaccine. Increasing biosafety tests for LAV prototypes might be mandatory in the future if they are to be commercialized. The results recently obtained with E75CV1 [15] and also observed with the OURT88/3 ASFV attenuated strain [12], allow the assumption that the use of specific pathogen free (SPF)-pigs could become an ideal in vivo model for evaluating the biosecurity of future live attenuated ASFV as vaccines. Infection of SPF-pigs with the intermediate optimal dose of E75CV1, albeit safe for farm pigs, resulted lethal for 100% of the SPF-animals [15]. The reasons behind the enhanced sensitivity of SPF pigs to attenuated ASFV strains could also partially explain the vaccine escapes observed under field conditions in the past [13]. The existence of residual virulence in ASFV attenuated strains has been described not only for naturally attenuated strains, such as E75CV1 and OURT 88/3 [12], but also for modified LAV generated by targeted depletion of virulent factors [39] and on many occasions this becomes complicated due to secondary infections attributable to the immune suppression induced by the virus.

With the aim of understanding the molecular mechanisms involved in attenuation of E7CV1, we are currently in the process of sequencing E75CV1. The availability of the E75 complete sequence (GenBank: FN557520) should facilitate the identification of the molecular changes in the genome of E75CV1 in comparison to its closest relative, thus contributing to better define virulence factors. Much effort has been made in the past with distantly related virulent and attenuated viruses and more recently, with related strains with different virulence facilitating the comparison [40]. These results have recently been extended to a more refined study helping to unmask the genome changes occurring during the adaptation of the virulent Georgia 2007 ASFV strain to Vero cell and subsequent attenuation [41].

We believe that performing comparative pathogenesis studies with two homologous strains with a unique origin, further validates the results obtained. Quantitative PCR results demonstrated the differential transcription kinetics existing between the attenuated and the virulent ASFV-strains. The infection with E75 shows a transcription profile quantitatively and qualitatively different from E75CV1 early after the infection. Thus, compared with the 10 genes significantly up and down-regulated by day 1 pi with E75CV1, E75 only up-regulated 4 of them, namely: *IL*-*12p40*, *TGF*-*βR1*, *TNF*-*α* and *IL*-*21*; the last not being significantly regulated with E75CV1 infection. The reduced number of immune modulators triggered at day 1 pi might help explaining the failure of the innate immune system to detect and control the first rounds of virus replication, thus allowing the rapid spread of the virus to lymphoid tissues, blood and nasals secretions. The lack of early recognition (day 1 pi) of the E75 virulent strain by the immune system might correlate with the observed down-regulation tendency of CD163, a putative ASFV receptor [42]. Conversely to what has been shown for E75CV1 the progression of the E75-infection resulted in a dramatic imbalance of the immune system by day 7 pi, with the significant up-regulation of 9 genes, namely: *IL*-*5*, *IL*-*1β*, *IL*-*6*, *IL*-*8*, *IL*-*10,IL*-*21*, *IL*-*23*, *DEFB2* and *TLR*-*3*. Surprisingly, the *TLR*-*3* gene was significantly and exclusively up-regulated at day 7 pi, perhaps reflecting the incapability of the *TLR*-*3* antagonist present in ASFV [43] to keep the *TLR*-*3* signaling pathway under control any longer. TLR-3, an Innate Pattern Recognition Receptor (IPRR) has been described as an efficient virus-sensor that could trigger friendly or unfriendly transduction cascades. The number and level of genes up-regulated at day 7 pi is unexpectedly high taking into account the damage suffered by the gastrohepatic lymph node, showing an almost unrecognizable architecture with massive cellular destruction. The dramatic increase found in sera of almost all the immune mediators tested: TNF-α, IFN-α, IL-12, IL-1β and sCD163 confirm the soluble factor storm suffered by acutely infected pigs in the latter phases of infection, most probably reflecting the massive tissue destruction found. Therefore, the dramatic activation of the immune system at day 7 pi might reflect the massive and disseminated fatal infection rather than a defensive mechanism. Unfortunately, the late detection of significantly high levels of sCD163 in sera at very late times makes its future use as an early diagnostic marker for ASFV unlikely. The presence of sCD163 in sera could however give new insight into understanding ASFV pathogenesis, perhaps playing an immunoregulatory role.

Conversely, the same viral dose of E75CV1 provoked the immediate activation of the immune system, reflecting most probably an efficient priming of the innate immune system, a key event for further mounting an efficient adaptive immune response, detectable at late time points post-infection. A careful dissection of the transcription profile by day 1 pi in tissues from E75CV1-infected pigs, allowed the confirmation of the significant up-regulation of *CD163*, *IL*-*1β*, *IFN*-*γ*, *IL*-*5*, *IL*-*6*, *IL*-*10*, *IL*-*12p40*, *TNF*-*α* and *TGF*-*βR1* and the down-regulation of *DEFB2*, immediately after E75CV1 infection. The up-regulation of CD163 has been associated with monocytes maturation into macrophages, and fits with the ability of ASFV to interact with the surface CD163 for viral entry [42]. The role of CD163 as receptor for ASFV entry has been proposed but not universally supported since reports demonstrated efficient infection of macrophages that do not express CD163 [44]. Regardless of this, the virus interaction with CD163 and other cell surface molecules most probably trigger the overexpression of the genes coding several pro-inflammatory cytokines: *IL*-*1β*, *IFN*-*γ*, *IL*-*5*, *IL*-*6*, *IL*-*12p40* and *TNF*-*α*; and also some anti-inflammatory mediators such as *IL*-*10* and *TGF*-*βR1* that might contribute to controlling the first rounds of virus replication while avoiding harmful inflammation. With the progression of the infection a more equilibrated immunological balance is observed, with 6 genes showing significant transcription up-regulation: *IFN*-*γ*, *IL*-*5*, *TNF*-*α*, *TGF*-*βR1*, *IL*-*21* and *IL*-*23* and 4 extra genes showing significant down-regulation: *DEFB1*, *CD163*, *IL*-*13* and *IL*-*18*. By day 31 pi, once the E75CV1 infection has been resolved and the memory specific B and T-cell responses have been established, a very tight regulation of the immune response was observed with only 2 genes being significantly up-regulated: *IL*-*23* and *NFκB*; and another two significantly down-regulated: *IL*-*1β* and *IL*-*4*, signature of Th 17-like responses. The continuous down-regulation suffered by the *IL*-*4* transcript reached its maximum level and significance at day 31 pi with E75CV1, coinciding with significant up-regulation of *IL*-*23* that in turn has been described to promote the Th17 switch [45]. This together with the continuous activation of *IFN*-*γ* and *TGF*-*βR1* throughout E75CV1 infection could also contribute to the continuous transformation of Th1 to Th17 cells, also previously described [46]. Th17 were first described as a CD4^+^ T-cell subset associated with chronic inflammatory processes, autoimmunity, allergy and transplant rejection [47]. Today we know that this is true if inappropriate, excessive, and non-specific Th17 effector responses are triggered, while on other occasions the induction of specific Th17/Th1 (IL-17^+^/IFN-γ^+^ cells) could exert beneficial effects. Thus, T(H)1/T(H)17 have been postulated as key cell subsets to fight against infections such as tuberculosis [48]. Thus, lacking (T(H)1/T(H)17)-cells has been described as a risk factor for the development of active tuberculosis in patients with HIV-1 infection and has been proposed as a useful biomarker in the development of tuberculosis vaccines. Further work will be needed to confirm the specificity and the role of Th17 cells in protection. The partial transcription profile shown here will be completed by performing complementary micro array and proteomic assays (work in progress).

As described in the introduction together with the innate immune responses, both specific B and T-cell responses play important roles in the protection afforded [30], these also being efficiently primed by our E75CV1 model. Unfortunately, all attempts to demonstrate the presence of specific antibodies capable of inhibiting the ASFV infection in vitro failed [28]. Similarly, the identical sequences of the E75 and BA71 haemagglutinin also failed to define the cross protection failure using a differential hemadsorption inhibition assay, as recently postulated for predicting the efficacy of ASFV vaccines [49]. Although no significant differences were observed by IFNγ-ELISPOT in response to the in vitro stimulation with either the homologous E75 or the heterologous BA71 viruses, we could not rule-out that these differences exist, as has been previously reported [12]. Technical limitations of our home-made IFNγ-ELISPOT assay performed with half a million PBMCs, did not allow a precise quantification of the specific spots found in response to each specific stimuli. Independent of these differences, one of the most relevant conclusions of the present work is the definition of a new in vitro correlation for ASFV protection to be added to the ones that already exist. The fact that CD4^+^ and double positive CD4^+^/CD8^low^ T-cells, defined as memory T-cells [30] indistinguishably proliferated in response to both homologous and heterologous virus fits with the IFNγ-ELISPOT data presented here. Conversely, the strong correlation between protection and in vitro recognition of strain-specific CD8^high^ T-cells, cytotoxic T-cells [30] is novel and might have important implications for the future design of vaccines. Cross-protective vaccines should be able to induce specific CTL responses against common antigens, an element that seems to be impaired in the LAV tested so far against ASFV. It has recently been published results demonstrating the establishment of a strong CD8^+^ T-cell immunodominance during ASFV recovery in vivo that could be overcome by prior immunization with subunit vaccines [30], a fact previously described for many pathogens. Several can be the reasons explaining the narrow repertoire of specific CD8^+^ T-cells induced, most probably being multifactorial and regulated by IFN-γ as explained for many other viruses [50]. The intrinsic mechanisms explaining ASF-immunodominance are being further studied with the aim of extracting useful lessons for future vaccine developments against ASFV.
